# Associations of maternal and placental extracellular vesicle miRNA with preeclampsia

**DOI:** 10.3389/fcell.2023.1080419

**Published:** 2023-02-22

**Authors:** Anat Aharon, Annie Rebibo-Sabbah, Rawan Sayed Ahmad, Ayelet Dangot, Tali Hana Bar-Lev, Benjamin Brenner, Adi Halberthal Cohen, Chen Ben David, Zeev Weiner, Ido Solt

**Affiliations:** ^1^ Hematology Research Laboratory, Department of Hematology, Tel Aviv Sourasky Medical Center, Tel Aviv, Israel; ^2^ The Sackler Faculty of Medicine, Tel Aviv University, Tel Aviv, Israel; ^3^ The Bruce Rappaport Faculty of Medicine, Technion, Haifa, Israel; ^4^ Department of Hematology, Rambam Healthcare Campus, Haifa, Israel; ^5^ Department of Obstetrics and Gynecology, Rambam Healthcare Campus, Haifa, Israel

**Keywords:** gestational vascular complications (GVC), extracellular vesicle (EVs), miRNA, placenta, preeclampsia, pregnancy-induced hypertension (PIH)

## Abstract

**Introduction:** Gestational vascular complications (GVCs), including gestational hypertension and preeclampsia, are leading causes of maternal morbidity and mortality. Elevated levels of extracellular vesicles (EVs), in GVC have been linked to vascular injury. This study aims to characterize placental and circulating EV miRNA in GVCs, and explores the involvement of EV-miRNA in GVC, and whether they may be used to distinguish between placental and maternal pathologies.

**Methods:** Blood samples were obtained from 15 non-pregnant (NP), 18 healthy-pregnant (HP), and 23 women with GVC during the third trimester. Placental sections were obtained after caesarian section. Platelet-poor-plasma (PPP) and EV pellets were characterized: EV size/concentration, protein content and miRNA expression were measured by nanoparticle tracking analysis, western blot, nano-string technology and RT-PCR. The effects of EVs on trophoblasts and EC miRNA expression were evaluated.

**Results:** Higher EVs concentrations were observed in HP-PPP and GVC-PPP (*p* < 0.0001) compared to the NP-PPP. The concentration of large EVs (>100 nm) was higher in PPP and EV pellets of HP and GVC compared to the NP group. EV pellets of pregnant women demonstrated lower expression of exosomal markers CD63/CD81 compared to NP-EVs. GVC-EVs expressed more human placental lactogen (hPL) hormone than HP-EVs, reflecting their placental origin. Screening of miRNAs in EV pellets and in PPP identified certain miRNAs that were highly expressed only in EVs pellets of the HP (13%) and GVC groups (15%), but not in the NP group. Differences were detected in the expression of hsa-miR-16-5p, hsa-miR-210, and hsa-miR-29b-3p. The expression of hsa-miR-16-5p and hsa-miR-210 was low in EV pellets obtained from NP, higher in HP-EVs, and significantly lower in GVC-EVs. Except for hsa-miR-29b-3p, which was upregulated in GVC, no significant differences were found in the levels of other miRNAs in placental sections. Exposure to GVC-EVs resulted in higher expression of hsa-miR-29b-3p compared to cells exposed to HP-EVs in villous trophoblasts, but not in EC.

**Conclusion:** Expression of hsa-miR-16-5p and hsa-miR-210 reflects maternal pathophysiological status, while hsa-miR-29b-3p reflects placental status. These findings suggest that EV-miRNA are involved in GVC, and that they may be used to distinguish between pathologies of placental and maternal origins in preeclampsia.

## 1 Introduction

Gestational vascular complications (GVC) are a group of pregnancy complications or syndromes associated with placental dysfunction, deficient utero-placental circulation, or intervillous and spiral vessel thrombosis. Due to various possible etiologies, a strict definition of GVC has not been established to date, but generally includes medical syndromes such as preeclampsia, intrauterine growth restriction, placental abruption, preterm labor, preterm pre-labor rupture of membranes, fetal demise/stillbirth, and recurrent abortions (Jung et al., 2022). GVCs are associated with significant maternal morbidity and mortality (Steegers et al., 2010; ACOG Practice Bulletin No, 2019). Clinical manifestations of GVCs may have a delayed presentation, only after a primary insult of abnormal trophoblast invasion in the uterine spiral arteries during early gestation. For example, inherited and acquired thrombophilias are possible thrombogenic etiologies of GVCs (Brosens et al., 2011), while abnormal placentation has been identified as the initial trigger of maternal endothelial dysfunction underlying preeclampsia (PE) (Gilani et al., 2016). While all possible etiologies of these obstetric syndromes have yet to be determined, they all present with a characteristic long preclinical stage, placental as well as fetal involvement, adaptive clinical manifestations, gene–environment interactions, and gene–gene interactions involving both maternal and fetal genotypes (Brosens et al., 2011; Jung et al., 2022).

Extracellular vesicles (EVs) have been considered as potential biomarkers of vascular injury, pro-thrombotic states (Aharon et al., 2009), and pro-inflammatory conditions related to PE and pregnancy-induced hypertension PIH (Germain et al., 2007; Kohli et al., 2016).

Small and large EVs (exosomes <100 nm and microvesicles >1 µm, respectively, per MISEV18 guidelines) (Thery et al., 2018), function as messengers promoting intercellular crosstalk (van Niel et al., 2018). Hypoxia-induced small EV proteins regulate proinflammatory cytokines and systemic blood pressure in pregnant rats (Dutta et al., 2020). In healthy-pregnant women, about 10% of circulating EVs are released from placental trophoblasts. The concentration of placenta-derived exosomes in maternal circulation increases continuously throughout the first trimester of pregnancy (Sakran et al., 2012), and the number of circulating exosomes increases by more than two-fold with gestational age (Salomon et al., 2014), EVs are important mediators of maternal-placental crosstalk (Shomer et al., 2013). In PE, excessive shedding of syncytiotrophoblast-derived EVs may lead to endothelial dysfunction, monocyte activation, and an excessive maternal inflammatory reaction (Messerli et al., 2010), In previous studies on EVs in healthy-pregnant women, and women with GVC, we found that the cargo within EVs, and their effects on the functions of endothelial (EC) and trophoblast cells (proliferation, migration, apoptosis and signal transduction pathways), vary according to the pregnant woman’s physiological/pathological state (Shomer et al., 2013). Others demonstrated that maternal EVs and platelets promote preeclampsia *via* inflammasome activation in trophoblasts (Kohli et al., 2016). The current study was performed in line with these studies and aimed to understand the underlying mechanisms responsible for these effects.

MiRNAs are small non-coding RNA molecules that regulate gene expression (O'Brien et al., 2018) and are involved in a variety of diseases and conditions. miRNAs are critical in cell development, proliferation, and apoptosis and each miRNA controls hundreds of target genes. EVs serve as the main transport vehicles for miRNAs and are a novel mechanism of genetic exchange between cells (Liu et al., 2019).

GVCs may be associated with alterations in miRNA expression in placental tissue and maternal circulation (Hornakova et al., 2020). Specifically, in pregnant women, circulating EV-miRNAs participate in maternal-fetal communication (Sarker et al., 2014; Foley et al., 2021) and placental miRNAs are released to the maternal circulation throughout pregnancy (Mouillet et al., 2015). Several miRNAs have been identified as involved in trophoblastic invasion, proliferation, and apoptosis (Cirkovic et al., 2021). Hypoxic conditions typical of early placentation affect miRNA expression in trophoblast cells (Pineles et al., 2007) and may explain the role of miRNA in development of PE and PIH (Truong et al., 2017). While previous studies have primarily focused on circulating exosomal miRNAs (Li et al., 2020), data on miRNA packed into “large” EVs is lacking. Moreover, the maternal and placental pathophysiologies underlying EV release have not yet been determined.

In order to define upstream regulators related to GVCs, the current study explored the involvement of miRNAs in EVs and in placental tissue in women with GVCs, and explored the effects of EVs on miRNA expression in trophoblast and endothelial cells.

## 2 Materials and methods

### 2.1 Participants and collection of samples

The study was conducted from 2014 to 2020 and approved by the institutional review board of the Rambam Healthcare Campus (Approval No. 2030) in Haifa, Israel. All participants signed informed consent. Blood samples were collected from non-pregnant healthy women (NP), and from 2 groups of pregnant women: healthy-pregnant (HP) and women with GVC, classified according to the guidelines of the American College of Obstetricians and Gynecologists (ACOG Practice Bulletin No, 2019) (Supplementary Figure S1).

### 2.2 EV isolation and characterization

#### 2.2.1 EV isolation

Blood samples were obtained during the third trimester of pregnancy, collected in EDTA and citrate tubes. Differential centrifugations were performed according to the current gold standard for EV isolation (MISEV 2018). Briefly, immediately following collection, blood samples were separated by two consecutive centrifugations (1500 g, 15 min, room temperature). Platelet-poor-plasma (PPP) was stored in aliquots at −80°C. Several studies demonstrated storage at −80°C and single freeze–thaw cycles were found not to have significant effects on either EV number or size (Lorincz et al., 2014; Yuana et al., 2015). To confirm that freezing did not affect EV concentration or size, Nanoparticle tracking analysis (NTA) was performed twice on selected samples that were defrosted at two different time points. We found that the concentration of EVs in PPP samples (particles/ml) remained constant throughout the freezing period (Supplementary Figure S2). Thus, all EVs were analyzed using thawed samples. EV pellets were isolated from equal volumes of PPP (250 µl) thawed samples. Citrate plasma is considered a better source for EV protein extraction (Palviainen et al., 2020). EVs of citrate plasma were also used for cell culture stimulation. Thawed EDTA PPP was used for RNA extraction, as recommended for Nanostring technology. Exosome isolation methods usually discarded 10,000 g pellet and continue with 100,000 g pellet (Coughlan et al., 2020). Recently, we emphasized the importance of the 20,000 g pellet fraction compared to samples that contained pellets of 100,000 g after discarding the 10,000 pellet (Aharon et al., 2021). In the current study, PPPs were centrifuged by MIKRO 220R, rotor 1189-A (Hettich) at 20,000g, 1h, 4°C; max acceleration, zero declaration.

Size and concentration of EVs were measured by transmission electron microscopy (TEM) and (NTA) [21].

#### 2.2.2 Transmission electron microscopy (TEM)

TEM analysis: 4 μl of EVs pellet samples isolated from NP, HP and GVC PPP were applied to formvar-carbon-coated, glow-discharged EM grids (EMS) and negatively stained with 1% uranyl acetate. Digital electron micrographs were acquired using a Thermo Fisher Scientific Tecnai T12 transmission electron microscope operating at 120 kV and equipped with a bottom mounted TVIPS TemCam-XF416 4k x 4k CMOS camera.

#### 2.2.3 Nanoparticle tracking analysis

NTA analysis, PPP samples, EV pellets and EVs in the supernatant, at the end of UC (EV sup.) of women from the different study groups were diluted in filtered PBS (0.02 µm) and subjected to static injection in the Nanosight instrument (NS300, Malvern Instruments Ltd, United Kingdom). For each sample, five consecutive videos were taken and further analyzed by the NTA software, with a threshold set at 5. Temperature was monitored throughout the entire recording time.

#### 2.2.4 Western blot

For western blot (WB) analysis, equal amounts of EV pellet (30-50 ug, measured by Pierce BCA protein assay kit, CAT 23227) obtained from similar PPP volumes (250 ul) were combined with a 2xlysis buffer (RayBiotech) supplemented with 1% proteinase inhibitor and 1% phosphatase inhibitors (Sigma) containing *β*-mercaptoethanol (1:20, Biorad). Samples were loaded and separated on 4%–20% Mini-PROTEAN TGX Precast Protein Gels (Bio-Rad) and then transferred to Trans-Blot Turbo Mini 0.2 μm Nitrocellulose Transfer Packs (Bio-Rad). The membranes were stained with Ponceau S solution (P7170, sigma) to ensure that proteins transferred from the gel to the membrane (Supplementary Figure S3), were washed and immunoblotted with the appropriate antibodies against exosome markers. Mouse monoclonal anti human-CD63 (ab59479) and CD81 (ab79559) were both used in 1:1000 dilutions. Anti-rabbit, anti-human placental lactogen hormone (hPL) (ab137099; 1;25,000 dilution) and Calnexin (ab10286, 1;10,000 dilution) endoplasmic reticulum (ER) protein, not expected to be enriched in EVs, serve as negative control (all from Abcam, USA). Secondary antibodies (anti-mouse 115-035-146 and anti-rabbit 111-035-144, both in 1:5000 dilution) were purchased from Jackson ImmunoResearch (PA, USA). The blot was imaged and quantified by myECL™ Imager and analyzed by My Image Analysis Software (both from Thermo Fisher Scientific, Waltham, MA USA).

### 2.3 RNA extractions and miRNA screening

#### 2.3.1 EV RNA

Was isolated from representative plasma samples (1 ml PPP) and EV pellets obtained from each study group. The miRNeasy isolation kit (Qiagen, Hilden, Germany) was used, with some modifications as previously described (Aharon et al., 2020; Levin et al., 2021). RNA concentration and quality were measured by a NanoDrop 2000 spectrophotometer (Thermo Fisher Scientific, Inc., Waltham, MA, USA). miRNA was screened by Nanostring platform by an external service (NanoString Technologies, Seattle, WA). Raw data on expression of 800 miRNAs were analyzed with the solver software (http://www.nanostring.com/products/ nSolver) (Rodosthenous et al., 2016). The nCounter assay was performed on 100 ng RNA of each sample, including six positive controls, six negative controls, five housekeeping genes (ACTB, B2M, GAPDH, RPL19, RPLP), and Non-Mammalian Spikes in miRNA probes (ath, miR159a,cel-miR-248, cel-miR-254, osa-miR414, osa-miR442).

#### 2.3.2 Placental sections

Were obtained from a representative number of women belonging to each of the pregnant groups, either during cesarean sections or immediately after delivery. For villous RNA studies, dissections of the chorionic plate were collected from three distinct locations on the placental surface as recommended (Roberts et al., 2019), submerged, and stored in RNA Later Solution (ThermoFisher Scientific). RNAs were isolated as previously described (Aharon et al., 2005), using Tri-reagent (Sigma-Aldrich Israel LTD) following a standard procedure.

### 2.4 Cell culture

To distinguish between maternal and placental involvement in miRNA expression, we exposed primary cell cultures from early stage human placenta villus trophoblast (HVT) to EV pellets that were obtained from the HP and GVC groups. The primary ECs representing the maternal side were also exposed to EVs of NP, as well as EVs of HP and women with GVC.

#### 2.4.1 Human early stage trophoblasts cells

Primary cells of the second trimester were purchased (Cat number 7120, ScienCell, Carlsbad, CA). As demonstrated in our previous study, EVs exert different effects on early stage trophoblast vs. term trophoblast cell cultures. Specifically, in HP women, treatment with EVs decreased apoptosis and induced higher migration in early-stage trophoblasts compared with untreated. Conversely, exposure to EVs obtained from women with GVCs increased term trophoblast apoptosis and inhibited early-stage trophoblast migration when compared to cells exposed to HP EVs (Shomer et al., 2013). Because GVCs begin earliest at 20 weeks of gestation (Peracoli et al., 2019), we preferred to study the effects of EVs on early stage trophoblasts and therefore did not culture HVT from term placentas. HVT were grown in a recommended trophoblast medium (50%, catalog number 7121, Sciencell) supplemented with Dulbecco’s Modified Eagle Medium–high glucose (22%, Biological Industries, Israel), F-12 (HAM) nutrient mixture (22%, Biological Industries, Israel), fetal calf serum (FCS) (10%, Biological Industries, Israel), and penicillin G sodium salt (10,000 units/mL) - streptomycin sulfate (10 mg/ml)–nystatin (1,250 units/mL) solution (1%, Biological Industries, Israel). The cells were grown at 37°C, 5% CO2. Passages 4-8 were used for experiments. HVT cell cultures were labeled with anti-hPL to ensure quality control of their contents. As in our previous studies, greater than 90% labeling with anti-hPL was considered a pure trophoblast culture (Shomer et al., 2013).

#### 2.4.2 Primary human umbilical vein ECs

Were isolated as previously described (Gao et al., 2019) [28]. HUVECs were labeled with anti-CD31^+^ to ensure quality control of their contents. Greater than 90% labeling was considered a pure EC culture (Tsimerman et al., 2011).

### 2.5 EVs effects on cell culture

#### 2.5.1 EVs cells interaction

EV pellets from 3 samples derived from each study group (HP and GVC) were labeled with pkh67 green fluorescent cell linker (MINI67-1 KT SIGMA) as described (Hazan-Halevy et al., 2015). Labeled EV samples were washed and concentrated with Amicon^®^ Ultra-15 Centrifugal Filter Units—10,000 NMWL (MERK) and then by Zeba Spin Desalting Columns0.5 ml (Thermo Scientific). Pkh67 green EVs (0.3 × 10E10). HVT cells were seeded on a 24-well plate in a recommended trophoblast medium. When cells reached 100% coverage, 8 × 10E4, they were washed with PBS. Pkh67 green EVs were added to the cells in serum free media for 6 h. At the end of incubation, cells were washed twice and detached with trypsin. Internalization of EVs into trophoblast cells was quantified by flow cytometry and documented by ZOE™ Fluorescent Cell Imager (Bio-Rad).

#### 2.5.2 EVs effects on cellular miRNA

(HVT Cells and EC were exposed to EV pellets (obtained from 250 ul of PPP) for 6 h in medium without-serum. Cells were washed and total RNA was purified with TRI reagent according to the manufacturer’s instructions. The purity and concentration of the RNA was evaluated by ultraviolet absorption at 260 nm and 280 nm (NanoDrop). cDNA was constructed using 50 ng of total RNA. Pools of five specific miRNA primers were prepared (Applied Biosystems) and their expression on treated compared to non-treated cells was validated.

### 2.6 miRNA validation by real-time polymerase chain reaction

All the reagents for these experiments were purchased from ThermoFisher Scientific (formerly Applied Biosystems). cDNA was synthesized with Taqman MicroRNA Reverse Transcription Kit and specific Taqman microRNA Assays. Different amounts of starting material were used depending on the nature of the sample (2 µl of RNA for circulating EVs or 50 ng of RNA from placentas, cells, or cell stimulated EVs). We developed a customized multiplex assay based on manufacturer’s guidelines for simultaneous amplification of several miRNAs. RT-qPCR was performed in duplicates per sample using TaqMan miRNA assays and Taqman Fast Advance Master Mix (Applied Biosystems). miRNAs from cell-samples and placental sections were normalized to U6 small RNA (Rice et al., 2015), while plasma EV samples were normalized to miR39 as recommended by the miRNeasy Serum Plasma Handbook, (QIAGEN). https://tools.thermofisher.com/content/sfs/manuals/cms_094060.pdf).

### 2.7 Statistical analyses

Data was analyzed using GraphPad Prism 5 software (CA, United States). Results were assessed by Kruskal Wallis one-way analysis of variance (ANOVA) and the Dunn’s multiple comparison test to compare the study groups. When only two groups were compared, non-parametric two-tailed Mann-Whitney U test student’s t-tests were used. *p* < 0.05 was considered statistically significant.

The results were expressed as mean ± SD. Effect size analysis was performed using Cohen’s d method in order to characterize the size of the differences between the groups. Small, moderate, and large effects were defined as 0.20, 0.40, and 0.80, respectively (Nunez Lopez et al., 2017; Peng et al., 2020). The exact numbers of each experiment performed (n) appears at the bottom of each graph and in the table. Easy Fisher Exact Test Calculator was used to calculate a 2 × 2 contingency table. Only some of the samples were evaluated in each test due to low sample volume. All samples were presented for each experiment. The Spearman r correlation tests were performed to evaluate the relation between disease severity (Systolic BP) and EV miRNA expression. *p*-value (two-tailed) < 0.05 was considered significant.

## 3 Results

A total of 56 women were enrolled. Patient characteristics including age, gestational week at sampling, and blood pressure are summarized in Table 1 and in the Supplementary Figure S1. The participants consisted of three groups: 15 non-pregnant (NP), 18 healthy-pregnant (HP), and 23 women with GVC. The ages were similar between the study groups. The mean number of gestational weeks at sampling was significantly less in the GVC than in the HP group. The mean blood pressure was higher in the GVC than in the HP group, indicating disease severity in the former. Uricemia (mg/dl) and proteinuria excretion (mg/24 h) were measured only in pregnant women with systolic BP above 140 mm Hg.

**TABLE 1:** Clinical Characteristics of non-pregnant and pregnant woman.

Parameter	Clinical Characteristics of Pregnancies for Placentas Studied			
	Non-pregnant women (NP), N = 15	Healthy-pregnant women (HP), N = 18	Women With Gestational Vascular Complications (GVC), N = 23	Statistical significance
Maternal age (years)	33.31 ± 6.750	33.50 ± 5.254	31.95 ± 5.317	NS
Gestational age (weeks)	NA	36.16 ± 3.154	34.40 ± 3.249	HP vs. GVC, p = 0.0264
Blood pressure, systolic (mm Hg)	114.8 ± 10.23	112.8 ± 12.85	156.4 ± 17.03	NP vs. HP, NS
				NP vs. GVC, p < 0.001
				HP vs. GVC, p < 0.001
Blood pressure, diastolic (mm Hg)	76.36 ± 9.137	64.90 ± 9.727	98.79 ± 9.193	NP vs. HP, p < 0.01
				NP vs. GVC, p < 0.001
				HP vs. GVC, p < 0.001
Uricemia mg/dl	NA	NA	5.96 ± 1.344	
Proteinuria - an excretion per 24 h	NA	NA	300–1000 mg, n = 13	
			>1000mg, n = 10	
Drug			*Labetalol, n = 4	
			*Eltroxin, n = 1	

*Labetalol, a beta-blocker - used to treat hypertension.

*Eltroxin - used to treat hypothyroidism.

NS, not statistically significant.

### 3.1 Characterization of EV size and concentration

**FIGURE 1:**
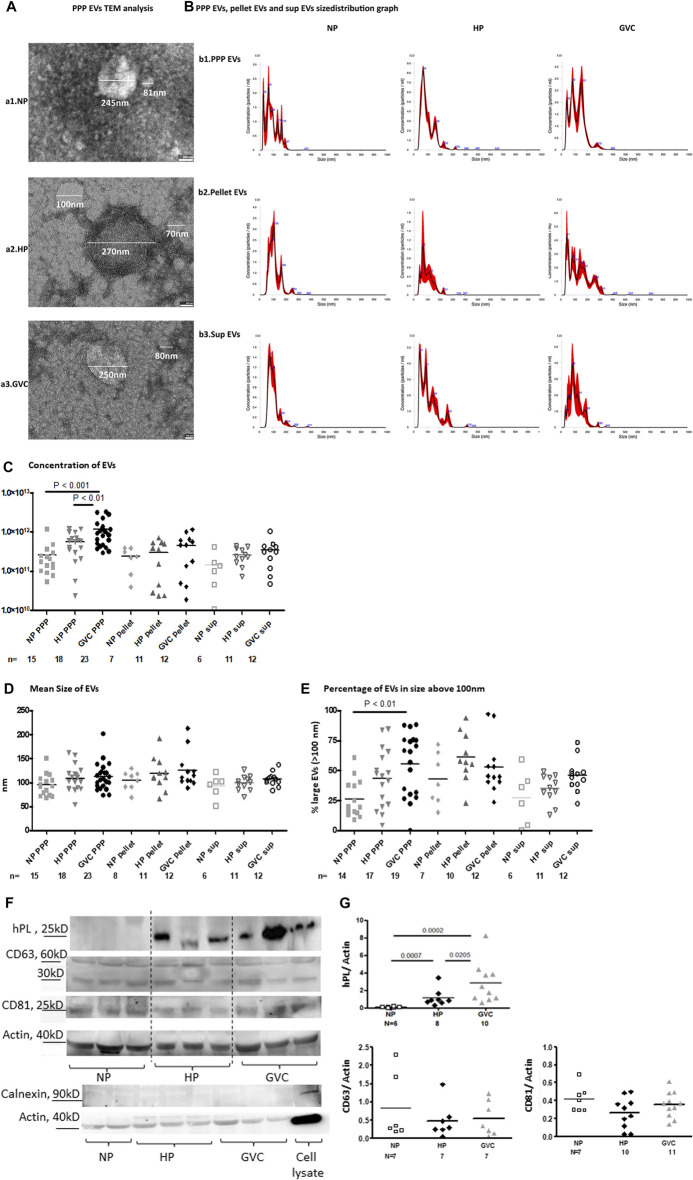
(Continued).

TEM images indicate EV heterogeneity and revealed that the sample pellets of the three study groups contained EVs of varied sizes (Figure 1A). Using NTA method, we analyzed the concentrations and size distributions of vesicles per mL of i) PPP, ii) EV pellets and iii) EV supernatant (sup.) Figure 1B presented representative graph distributions of each of the study group samples (PPP, pellet and sup). Significantly higher EV concentrations were observed in PPP of the HP (5.64e+11 ± 3.66e+11 EVs/ml, *p* < 0.01) and GVC (1.17e+12 ± 9.14e+11 EVs/ml, *p* < 0.001) groups than in PPP of the NP group (2.6e+011 ± 2.8e+11 EVs/ml) (Figure 1C).

The mean EV size was significantly greater in the GVC-PPP than the NP-PPP group (112. 7 ± 28.43 nm vs. 95.16 ± 22.72nm; *p* = 0.0316, with size effects differences Cohen’s d = 0.681) (Figure 1B). The concentration of large EVs (>100 nm) was found to be higher in GVC-PPP samples (55.33 ± 26.44%) compared to their concentration in the NP-PPP (26.42 ± 15.77%, *p* < 0.01, Cohen’s d = 1.328) and HP-PPP (43.52 ± 24.34%, *p* = 0.1206 (NS), Cohen’s d = 0.834) groups (Figure 1C). The percentage of large EVs (>100 nm) on EV pellets of HP and GVC pellets were two times higher than in their associated supernatants (HP pellet vs. sup. Cohen’s d = 0.698,196; GVC pellet vs. sup. Cohen’s d = 1.55467). EV pellets of pregnant women showed similar expression of exosome markers compared to NP EVs (Figures 1F, G). Size effect analysis displayed moderate change in CD81 expression between HP-EVs and GVC-EVs (Cohen’s d = 0.7385). In addition, size effect analysis display a moderate change and higher hPL expression in EVs obtained from the GVC group than EVs obtained from the HP group (Cohen’s d = 0.510,164). These findings may indicate that more hPL was packaged within the EVs released from placenta trophoblast cells of this group. Calnexin was used as a negative control marker of the endoplasmic reticulum (ER appear only in cells lysate and were absent t in EVs samples (Figures 1F, G).

### 3.2 EV miRNA screening and validation

#### 3.2.1 EV miRNA screening

Double sets of screening using NanoString technology were performed using patient EV samples. We compared miRNAs in EV pellets and miRNAs in PPP (consisting of EVs and free miRNA) of the same sample. Screening identified 104 miRNAs with a significant copy number (>100) in at least one of the samples obtained from EV pellets or in PPP.

**FIGURE 2:**
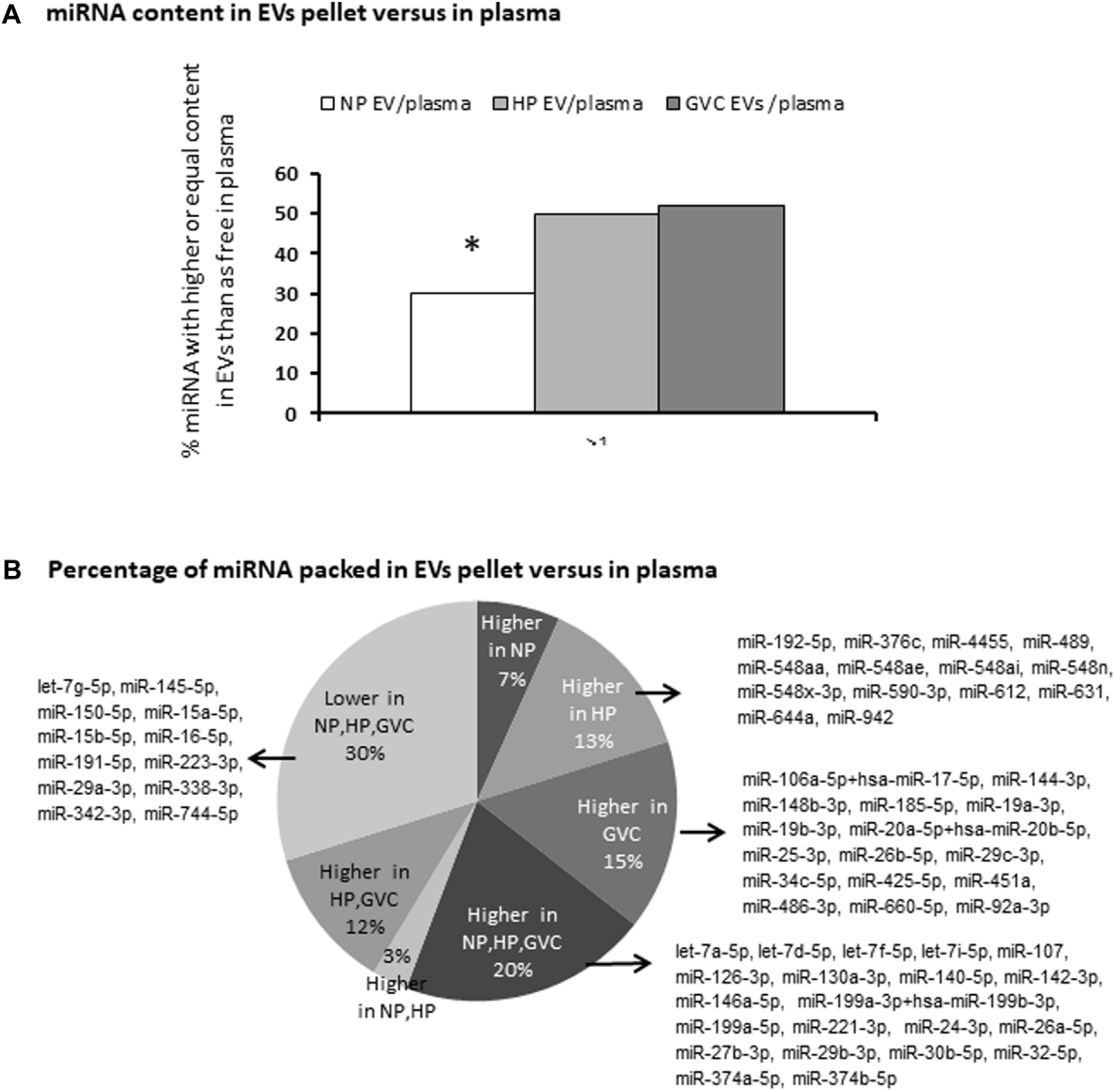
Screening to identify differences in the expression of miRNAs between those packed in extracellular vesicles and those circulating in plasma miRNA was profiled using the NanoString platform and analyzed with nSolver Software 3. The percentage of miRNA with higher or equal content in EV pellets compared to PPP (*p ≤ 0.05 Easy Fisher Exact Test Calculator). **(A)** The proportion of miRNAs packed in EVs versus PPP and the list of miRNAs that were more highly expressed only in the healthy-pregnant (HP) group, only in the gestational vascular complications (GVC) group, and in both pregnancy groups (HP and GVC), respectively. The proportion of miRNAs found to be “packed” in EVs is higher in all three study groups (NP, HP, and GVC), or lower in all three groups **(B)**.

Expression levels of PPP miRNAs and miRNAs in EV pellets both differed between groups. In NP, only 30% of the miRNA was found in EV pellets; however, the HP and GVC groups had significantly higher rates of miRNA in EV pellets, reaching 50% and 51%, respectively (Figure 2A). Moreover, among the 104 miRNAs with substantial expression, select miRNAs were highly concentrated only in either the EV pellets of the HP group (13%) or the GVC group (15%), but not the NP group (Figure 2B). For EVs pellet of HP and GVC, two samples of each group were screened and compared to EVs pellet of single NP sample screening data results presented in Supplementary Table S1

#### 3.2.2 Circulating EV miRNA validation

**FIGURE 3:**
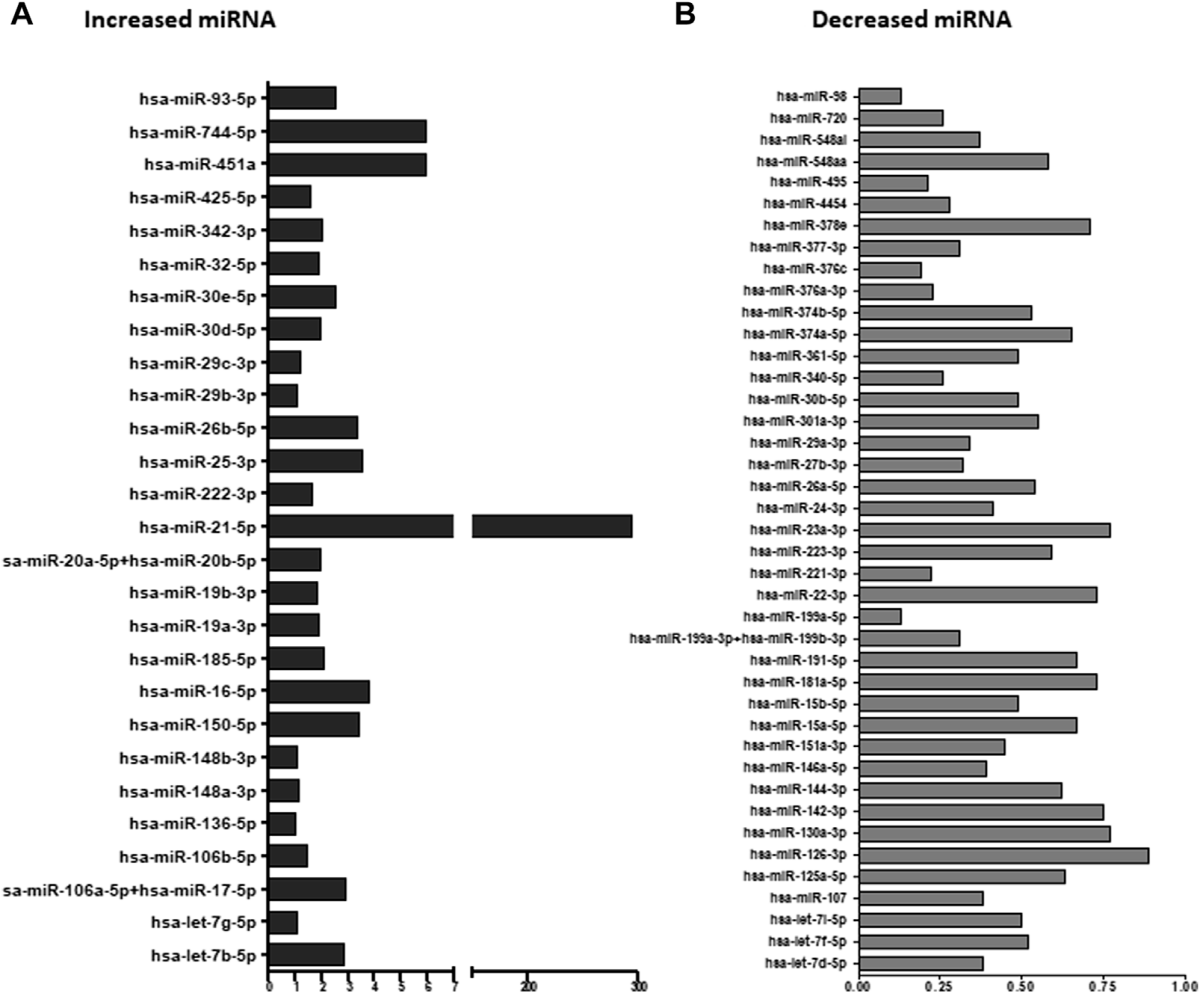
Ratio of extracellular vesicle (EV) miRNA from women with gestational vascular complications (GVC) compared to healthy-pregnant women (HP). EV pellet miRNA was profiled using the NanoString platform and analyzed with nSolver Software 3. The graphs present the ratios of Average EV miRNA from two samples of women with GVC compared to average EV miRNA from two samples obtained from HP women. miRNA expression was greater in EV pellets from the GVC than the HP group **(A)** and lower in EV pellets from the GVC compared to the HP group **(B)**.

Average screening results of EVs pellets obtained from two GVC samples compared to average of two HP samples revealed 20 specific miRNAs which were 50% more concentrated in EV pellets of the GVC compared to EV pellets of the HP group (Figure 3A). 23 other miRNAs, for which the expression was 50% lower in EV pellets obtained from the GVC samples compared to the HP samples, were found (Figure 3B). From all these miRNAs, we selected certain miRNAs (Supplementary Table S1) that have been identified in previous publications as important regulators of placental regulation and GVCs (Hornakova et al., 2020; Ali et al., 2021) to ensure that our study covered the most important miRNAs related to GVC.

Validation of EV pellet miRNA expression by RT-PCR was performed for each of the study groups. Expression levels of 21 selected miRNAs (Supplementary Table S1) were assessed.

**FIGURE 4:**
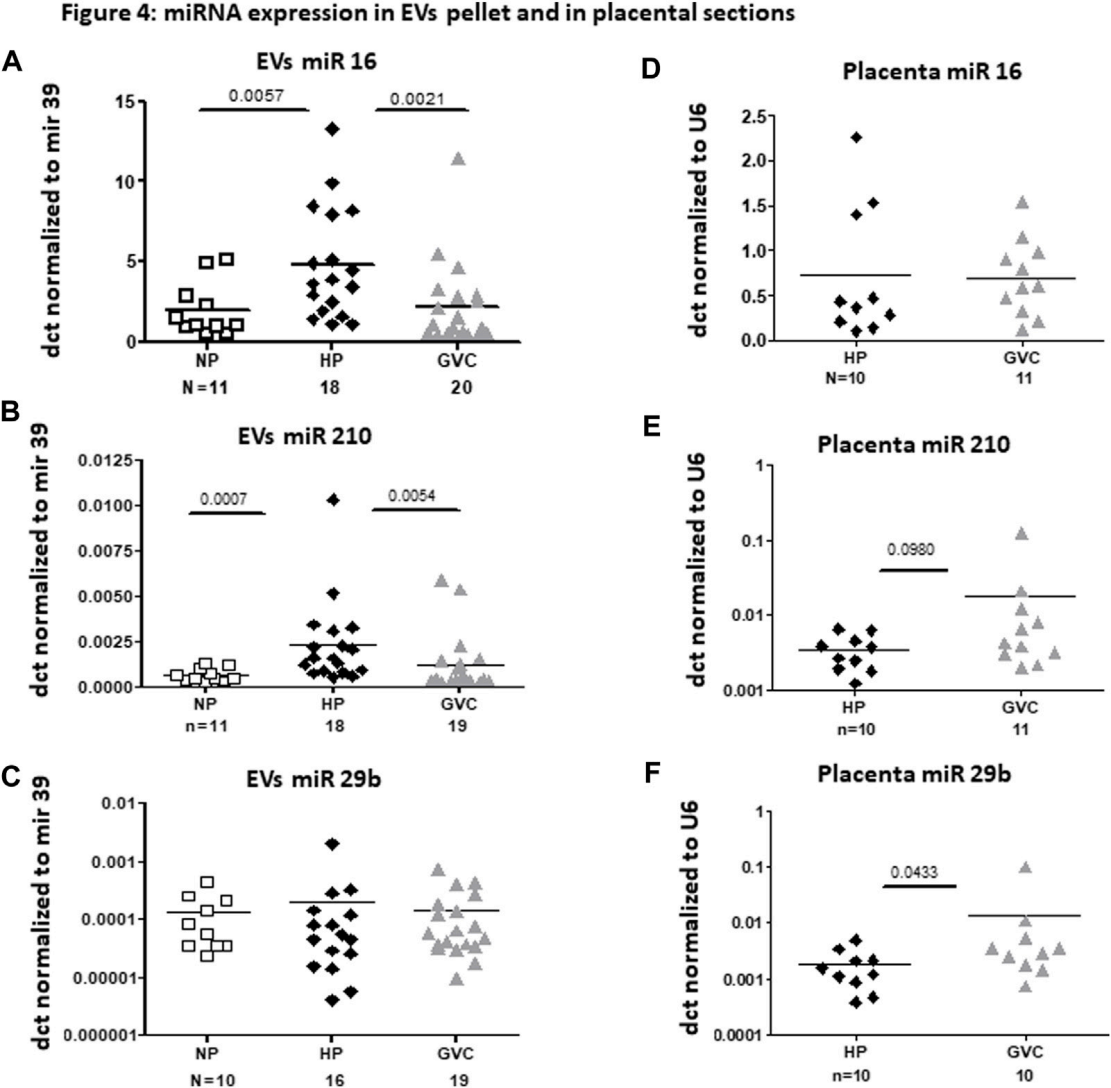
miRNA expression in EV pellet and placental sections from the study groups miRNA expression in EV pellets of the non-pregnant group, and EV pellets and placental sections of the pregnant groups [healthy-pregnant (HP) and gestational vascular complications (GVC)] were validated by real-time polymerase chain reaction. Each EV miRNA sample was normalized to cel-mir-39 spike-in. Each placental miRNA sample was normalized to U6, and expressed as dct. **(A–C)** present expression of the following miRNA EVs: hsa-miR16 **(A)**, hsa-miR210 **(B)**, and hsa-mir29b-5p **(C)**. **(D–F)** present expression of these miRNAs in placental sections: hsa-miR16 (4 days), hsa-miR210 **(E)**, and hsa-mir29b-5p **(F)**.

Significantly greater expression of EV miRNA, specifically of hsa-miR-16-5p (*p* = 0.0057, Cohen’s d = 1.047.) and hsa-miR-210 (*p* = 0.0007, Cohen’s d = 1.0248.), were observed in the HP compared with the NP group. In contrast, significantly lower expression of hsa-miR-16-5p and hsa-miR-210 was observed in EVs obtained from the GVC compared with that of the HP group (*p* = 0. 0021, Cohen’s d = 0.842 and *p* = 0.0054, Cohen’s d = 0.546, respectively) (Figures 4A, B). EV miR-29b-3p levels were found to be similar between the study cohorts (Figure 4C).

#### 3.2.3 Placental sections miRNAs

In most of the validated miRNAs, expression was similar in placental sections from the HP and GVC groups. The hsa-miR-29b-3p level was significantly higher in the placentas of women from the GVC compared with the HP group (*p* = 0.0433, Cohen’s d = 0.528) and trend of increase were found in hsa-miR-210 (*p* = 0.098, Cohen’s d = 0.541) (Figures 4C, D).

**FIGURE 5:**
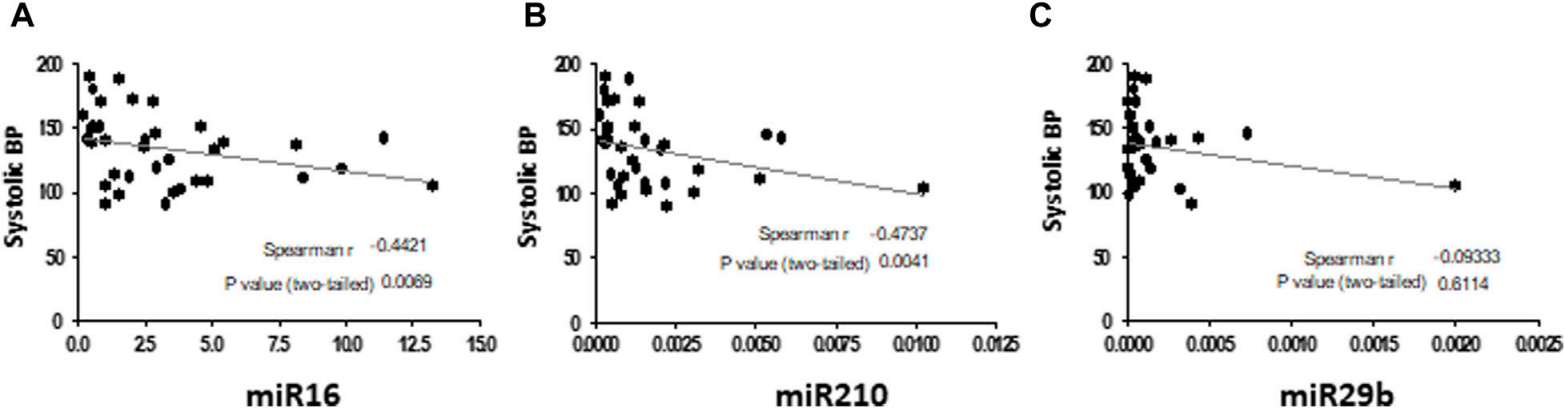
Correlations between systolic blood pressure (BP) in pregnant women (HP and with GVC) and EV pellet miRNA expression The graphs present Spearman test correlations analysis between systolic blood pressure in the pregnant women (the HP and GVC groups combined) and expression levels of EV miRNA16 **(A)**, miRNA210 **(B)** and miR29b **(C)**.

The GVC group was characterized by significantly higher systolic blood pressure (Table 1; Supplementary Figure S1), indicative of a pathological condition. Moderate negative correlations (spearman r = -0.4) were found between systolic blood pressure in the pregnant women (the HP and GVC groups combined) and expression levels of EV miRNA16 (*p* = 0.0069) and miRNA210 (*p* = 0.0041). No correlations were found between high blood pressure and expression of EV miR29b in pregnant women (Figures 5A–C).

### 3.3 EVs effects on cell culture

#### 3.3.1 Trophoblast cells interaction

**FIGURE 6:**
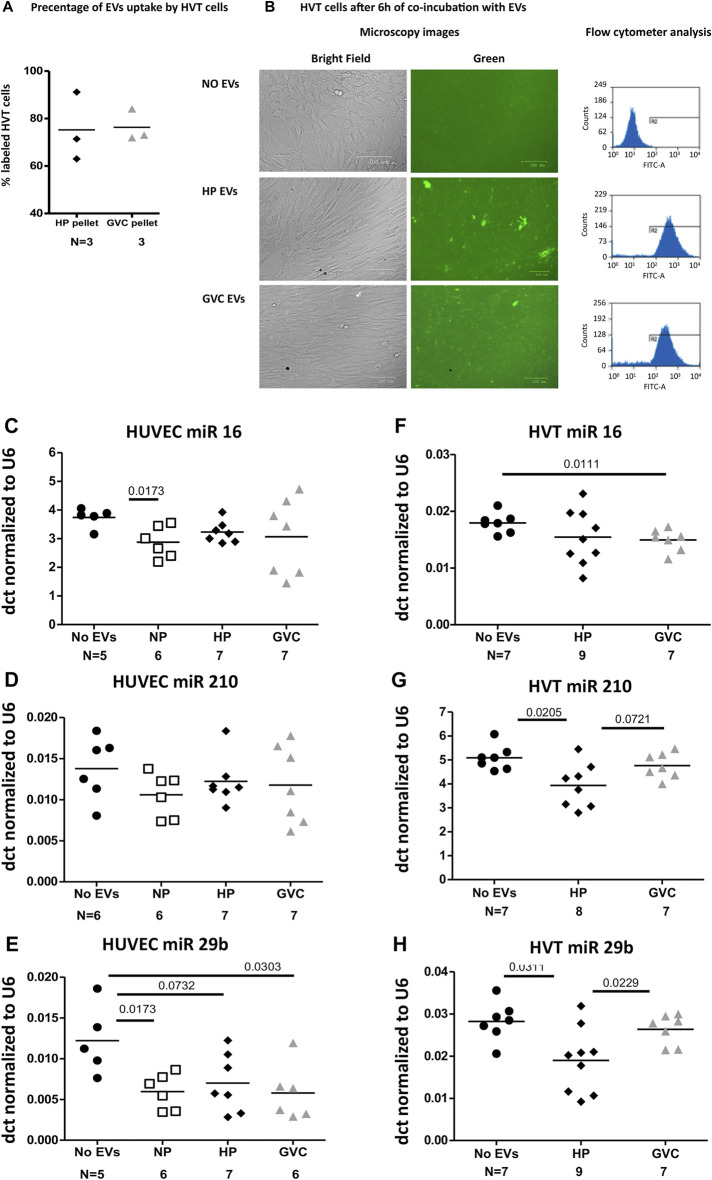
(Continued).

A similar rate of green EVs internalization trophoblast cells was found in all samples (HP-EVs 75.20 ± 14.48%, GVC-EVs 76.33 ± 6.749%). HVT exposure (6 h) to both groups of EVs (HP and GVC) did not affect viability or morphology of HVT cells (Figures 6A, B) confirming our previous study that showed that HP EVs decreased HVT cells apoptosis while GVC-EVs did not affect their viability compared to untreated cells (Shomer et al., 2013).

#### 3.3.2 Differences in EV pellet effects on miRNA expression in endothelial and trophoblast cells

The differences in the effects induced by EVs obtained from NP, HP, and GVC groups on miRNA expression in ECs and trophoblast cells were normalized to housekeeping gene U6 and expressed as the delta-Ct (dct). Exposure of EC cells to EVs of the study cohort did not affect cell expression of miRNAs hsa-miR-16-5p, hsa-miR-29b and hsa-miR-210 (Figures 6C–G). In contrast, Exposure of trophoblast cells to EVs obtained from women with GVC significantly increase the expression of hsa-miR-29b in trophoblast cells (*p* = 0.0229, size effect Cohen’s d = 1.230 and induce trend of increase in hsa-miR-16-5p (*p* = 0.072, Cohen’s d = 1.114,574). While the effects of HP EVs and GVC EVs were similar on hsa-miR-210 expression only GVC EVs significantly reduced its expression compared to non-treated cells (*p* = 0.011, Cohen’s d 1.617) (Figure 6H).

## 4 Discussion

This study is the first to identify specific miRNAs cargo in small and large EVs of pregnant women. However, we cannot definitively state whether miRNA decorates EV corona or is packaged within the vesicles. It also sought to define the differences in miRNA expression related to either maternal vasculature (circulating EVs, ECs) or placental sites (placental sections, trophoblast cells).

### 4.1 EV size, concentration and placental origin

Pregnant woman PPP (obtained from HP and GVC) contained higher concentrations of EVs compared to NP PPP, but only the GVC group displayed a significant increase in EV size. This correlated with higher concentrations of hPL indicative of a placental origin. Trophoblast cells are lined the placental villus, invade the decidual spiral arteries and are the only placental cells that come in direct contact with maternal circulation (Zhao et al., 2021). Therefore, we can assume that placental EVs in the circulation originated from trophoblast cells.

In the current study, we demonstrated the importance of the fractions enriched with “large EVs” obtained by 20,000 g. We also showed the qualitative differences in miRNA cargo contained in pellets of enriched samples with “large EVs,” compared with samples of PPP containing both miRNAs packaged within EVs as well as freely circulating miRNAs. The increase in particle size and concentration is an important determinant of the “cargo” they carry and can transport between cells. Analysis of the size distribution demonstrated that PPP and EV pellets contained large and small EVs in similar proportions. However, using a UC isolation method to analyze the sediment only extracted part of the EVs from the PPP samples, while some EVs (mainly small EVs) remained in the supernatant as previous described [30]. While most studies based their findings on exosomal miRNA and eliminated the fraction of large EVs, the current study focused on the advantages of using a composition of small and large EVs. We assume that miRNA in EV pellets were mainly packaged inside the vesicles, while in PPP samples, they also appear as free-form molecules. In the current study, we found more miRNAs packaged in EV pellets of pregnant women than in PPP. This suggests that certain miRNAs may be selectively packaged during pregnancy. Preliminary evidence (Turchinovich et al., 2013; Mitchell et al., 2015) suggests that the relevant mechanism is apparently a non-random process (Diehl et al., 2012; Boon and Vickers, 2013; Niu et al., 2018) related to EVs as a source of miRNAs that promote GVCs. Previously, we demonstrated that EVs from women with GVCs contain higher levels of pro-inflammatory cytokines, promote apoptosis, suppress migration of ECs and early-stage trophoblasts (HVT), and inhibit tube formation (Shomer et al., 2013).

### 4.2 The role of miRNAs in placenta versus maternal circulation

Of the numerous miRNAs that were screened and validated in this study, significant differences were found in three miRNAs: hsa-miR-16-5p, hsa-miR-210, and hsa-miR-29b-3p.

Several recently published articles have comprehensively reviewed the role of miRNAs in regulating placental and fetal development (Hornakova et al., 2020; Ali et al., 2021), suggesting that miRNAs promote dysregulation of placental development, and consequently, maternal physiology and fetal growth and development. These reviews found mir-210 to be one of most dominant. Fifteen different studies showed dysregulation of miR-210 in PE, and 3 studies showed its dysregulation in IUGR. Only one other study also reported the upregulation in placental mi-16 and miR-29b that we found in the current study. Our results support findings reported in Kyoto Encyclopedia of Genes and Genomes analysis (KEGG map, Supplementary Figure S4; Vlachos et al. 2015) that suggest the three miRNAs hsa-miR-16-5p, hsa-miR-210, and hsa-miR-29b-3p regulate 44 genes related to the PI3K-AKT signaling pathway. These miRNAs act as the first step in the cell regulation pathway in PE. Specifically, EV miR-16 and mir-210 were significantly higher in the HP than NP and GVC groups, correlating negatively with disease severity as indicated by elevated blood pressure. In contrast, mir-29b expression was similar across EVs but higher in placental sections and in induced HVT cells.

#### 4.2.1 miRNA-210

MiRNA210 is induced in response to hypoxia and regulates more than 900 genes related to placental development and placental pathologies (Ali et al., 2021). Elevated plasma levels of hsa-miR-210 have been found to correlate with PE severity (Xu et al., 2014; Biro et al., 2017), while detection of miR-210 in the urine positively correlates with the level of proteinuria (Gan ZL et al., 2017). Furthermore, increased expression of miR-210-5p has been documented in placentae of women with PE (Awamleh et al., 2019). Based on these findings, we decided to include this miRNA in the PCR validation, despite its low expression in the study screening results. In contrast to previous studies, our results showed significantly lower EV miR-210 levels in the GVC compared to the HP group. These findings were unexpected considering the increased levels of this miRNA that were found in blood and urine of PE patients. However, we did detect higher EV-miR210 expression in placental sections of women with GVC, consistent with previous studies (Luo et al., 2016). Discrepancies may be due to differences in the timing of sampling as well as the sources of RNA used to measure levels of miRNAs, both circulating in the blood and encapsulated inside of EVs. Exposure of trophoblast cells, which are the main source for placental circulating EVs, to GVC- EV pellets or to HP-EV pellets induces similar reductions of mir-210 expression compared to untreated cells. These results imply that trophoblast cells do not reflect the internal placental processes. It may be inferred that trophoblast EVs did not reflect the same expression as placental sections and the source of the decreased miRNA EVs in GVCs is primarily related to maternal pathology.

#### 4.2.2 miRNA-16

Low expression of miR-16 has been reported to correlate with fetuses that are small for gestational age (Maccani et al., 2011). Altered expression of miR16 in decidua-derived mesenchymal stem cells was related to the development of PE (Wang et al., 2012). The present study found low expression of miR-16 in GVC-EVs but did not discover any differences in placental miR-16 expression under the examined conditions. This supports the presumption that EVs with altered miR-16 expression originate from maternal cells.

#### 4.2.3 miRNA-29

The family of miR-29 microRNAs has been reported as possible regulators of more than 4000 gene products, with diverse roles in regulation of cell survival (Slusarz and Pulakat, 2015). miR-29b was found to induce apoptosis and inhibit invasion and angiogenesis of trophoblast cells (Li et al., 2013). Overexpression of miR-29b decreased cell proliferation of decidua-derived mesenchymal stem cells (Xin et al., 2017). In the current study, miR-29 expression was significantly higher in placental sections of the GVC group compared to the HP group. Moreover, GVC-EVs upregulated miR-29b in early stage primary placental HVT cells in culture. Previously, we demonstrated that EVs of women with GVCs contain higher pro-inflammatory cytokine levels, promote apoptosis, and suppress migration of HVTs. EVs potentiate these effects through an extracellular signal-regulated kinase pathway altering ERK signal transduction (Pineles et al., 2007). miR29b may be the missing “link in the chain” responsible for inducing placental dysfunction in GVCs.

#### 4.2.4 Summary

The relationship between the development of GVCs and changes in the expression levels of tissue-specific and circulating miRNAs has been demonstrated in several studies. miRNA packaging in vesicles membranous protect them from rapid degradation in circulation and enable their penetration to target cells. While the majority of the studies support the notion that EVs contain miRNA and actively transfer their miRNAs cargo between cells, a recent study claimed that delivery of different species of RNAs as well as proteins through the EVs is an extremely inefficient process (Albanese et al., 2021).

EV-miRNAs are involved in GVC development and reflect disease severity.

Our findings suggest that EV hsa-miR-16-5p and miR-210 of maternal origin, while hsa-miR-29b-3p is essentially of placental origin. These findings highlight the potential utility of these miRNAs to serve as biomarkers for distinguishing between the placental and maternal pathophysiologies underlying PE. Moreover, based on these results and those of our previous study, in which GVC-EVs induced apoptosis in HVT (14), we suggest that miR-29b serves as one of the main regulators of trophoblast cell viability.

The current study has some limitations. Previous studies reported increased blood uric acid levels during the third trimester of pregnancy. Uric acid levels of >5 mg/dl at term and proteinuria above 300 mg/24 h can be used as both clinical markers of GVC severity and tools to distinguish preeclampsia from PIH (Johnson et al., 2011; Le et al., 2019). In the current study, we did not divide the GVC group into mild and severe PE. However, GVC EVs samples were combined for analysis as a single group. Other limitations include obtainment of blood samples at delivery is a late stage of GVC disease, obtainment of placental samples from only about half of the pregnant women, relatively small cohorts sizes, and performance of certain tests only on specific samples. Additionally, the women classified with diagnoses of GVCs presented with heterogeneous disorders (PIH, mild or severe PE).

## 5 Conclusion

Numerous studies have linked different patterns of miRNA dysregulation to GVCs, supporting the role of placental EV cargo in disease progression. However, trophoblast EVs, the main source of placental EVs, do not always reflect placental pathophysiology and function. In this study, we emphasized the importance of the large EVs which are usually discarded, and identified specific miRNAs related to GVCs that may distinguish between pathologies of maternal (hsa-miR-16-5p and hsa-miR-210) and placental (hsa-miR-29b-3p) origins in preeclampsia.

## Data Availability

The original contributions presented in the study are included in the article/Supplementary Material, further inquiries can be directed to the corresponding author.
