# Supplementary figures and images for: Associations of maternal and placental extracellular vesicle miRNA with preeclampsia
Source: Front Cell Dev Biol. 2023 Feb 22;11:1080419. doi: 10.3389/fcell.2023.1080419 (PMC9992195; doi:10.3389/fcell.2023.1080419)

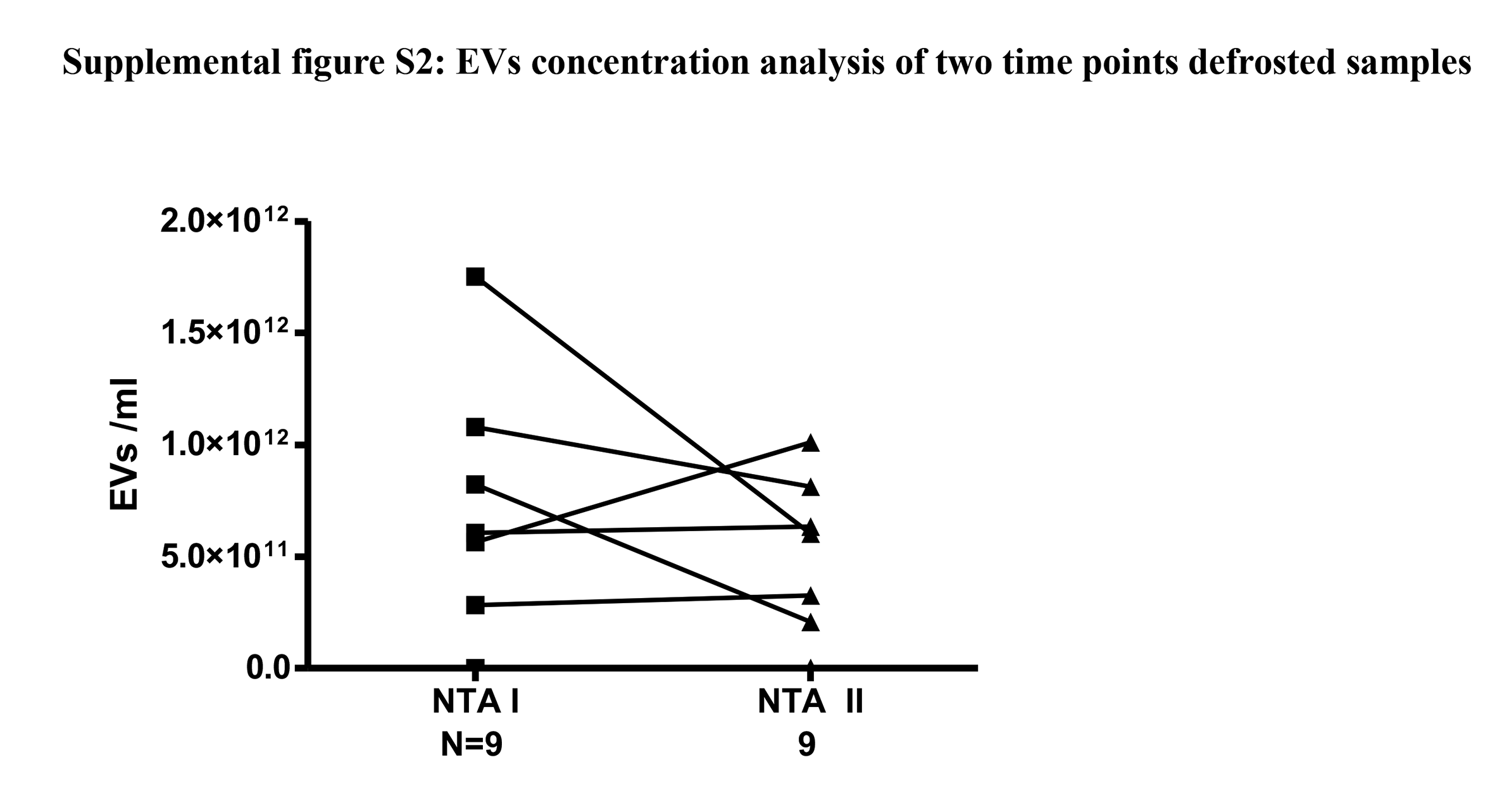

Supplement: Supplementary file 1 [file Image3.TIF]

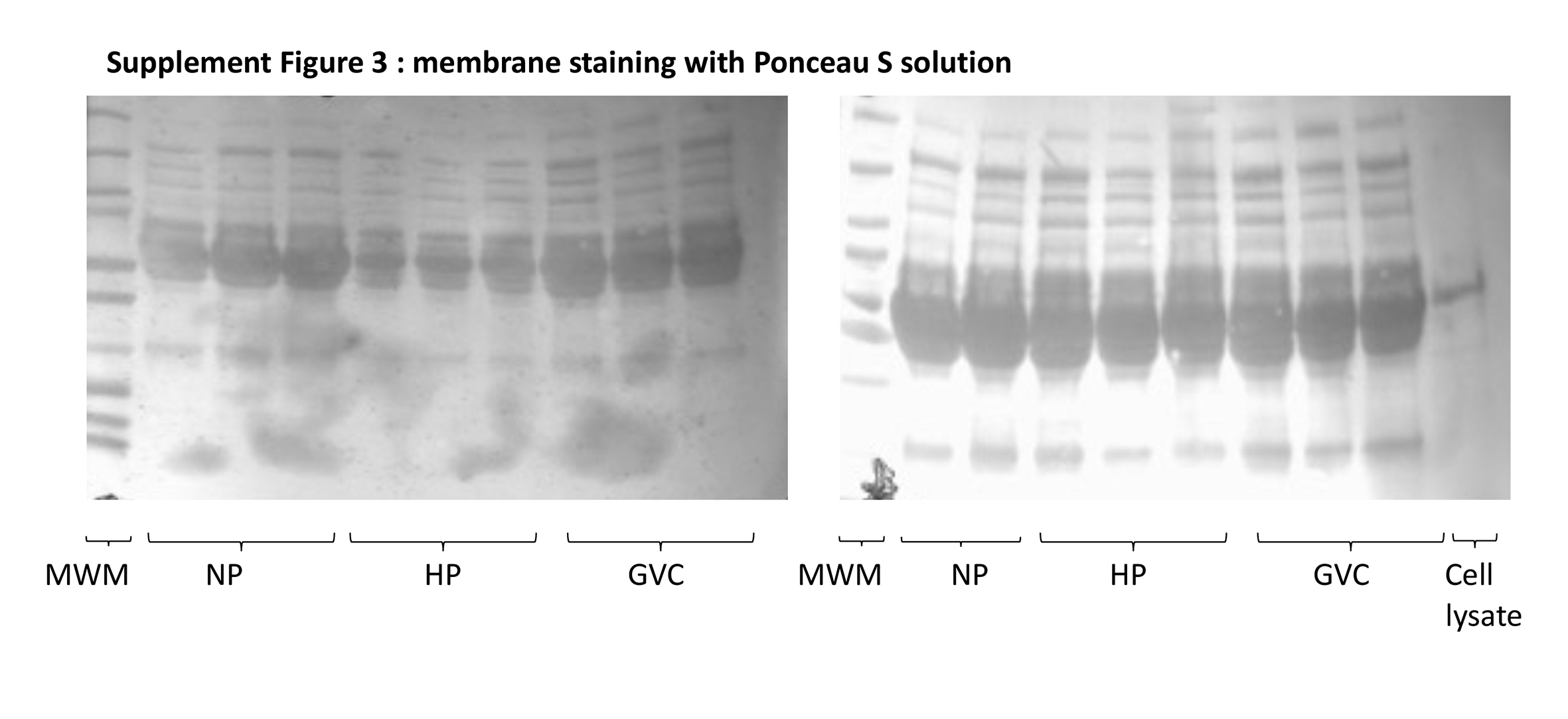

Supplement: Supplementary file 2 [file Image4.TIF]

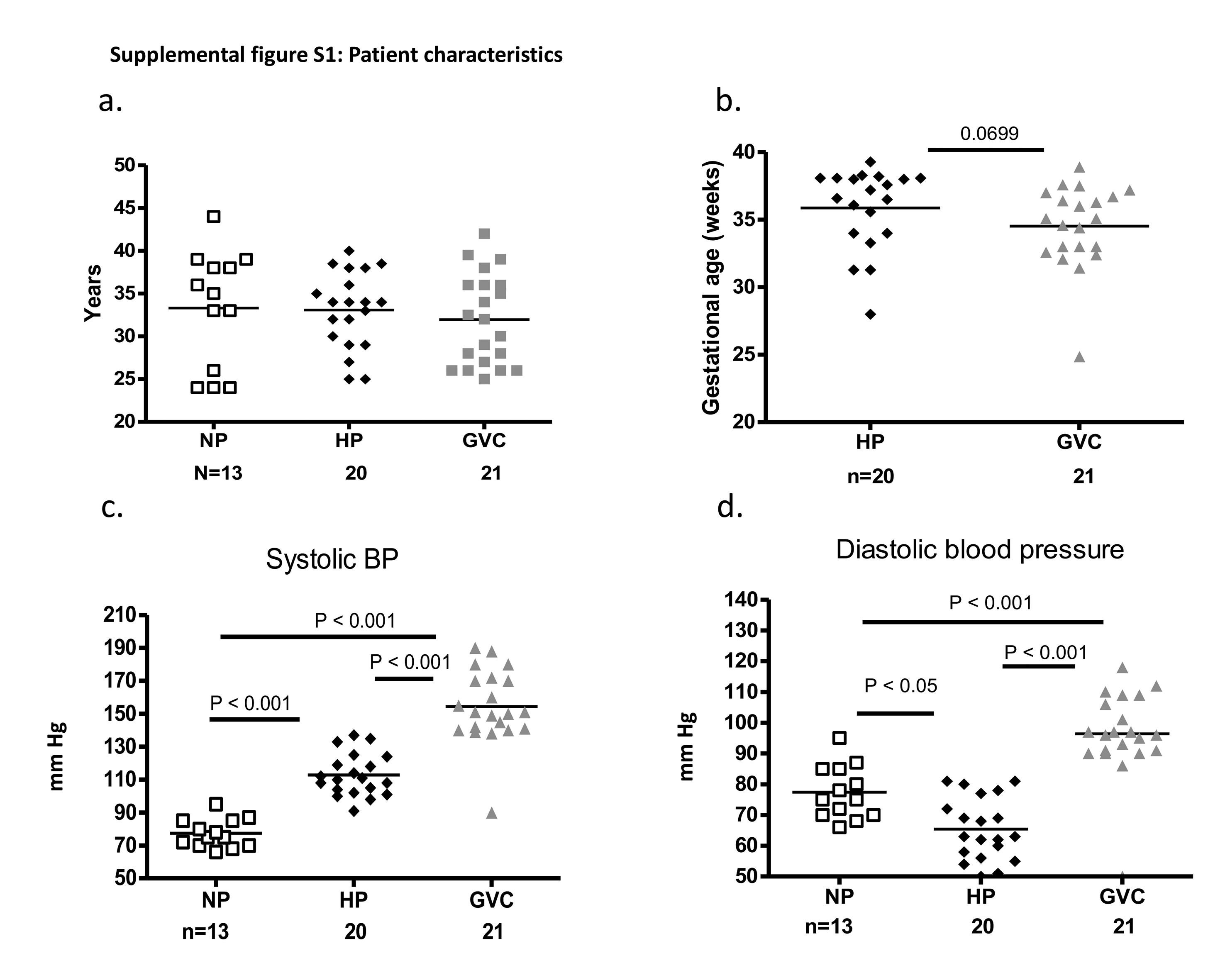

Supplement: Supplementary file 3 [file Image2.TIF]

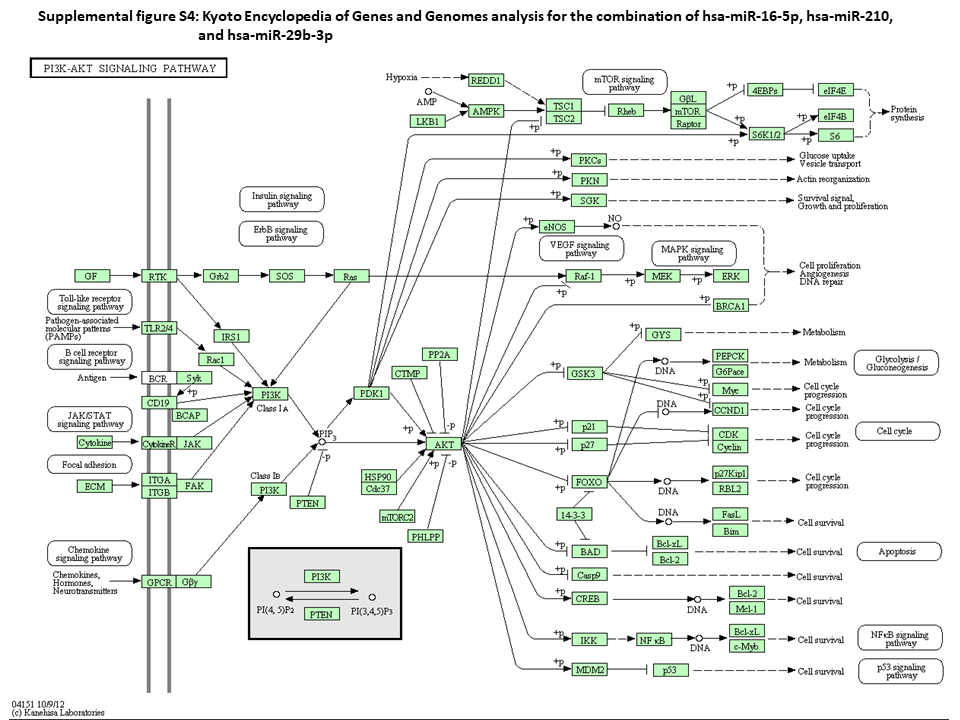

Supplement: Supplementary file 4 [file Image1.TIF]
